# Non-governmental organizations involvement on search and rescue operations in the Mediterranean Sea: friend or foe?

**DOI:** 10.3389/fpubh.2024.1362214

**Published:** 2024-10-17

**Authors:** Michele Salvagno, Alessandro Jachetti

**Affiliations:** ^1^Department of Intensive Care, Hôpital Universitaire de Bruxelles (HUB), Brussels, Belgium; ^2^Emergency Department, S.C. Pronto Soccorso, Fondazione IRCCS Cà Granda - Ospedale Maggiore Policlinico Milano, Milan, Italy

**Keywords:** NGO, migration, SAR, Mediterranean, public health

## Introduction

In the last two decades, the Mediterranean Sea has become a major global hotspot for migration, with thousands of individuals attempting to cross its waters in search of a better life in Europe. The timeless lines from renowned poet Cesare Pavese resonate today more than ever: “*I know not what world lies beyond this sea // But every sea has another shore, you see // And there I shall be.”*

Several Non-Governmental Organizations (NGOs) have started launching rescue missions in the Central Mediterranean Sea, operating under the framework of international legal obligations that mandate Search and Rescue (SAR) operations for those distressed at sea (1, 2). Nonetheless, these NGOs operate within a complex and disputed legal environment, governed by key international conventions such as the 1979 International Convention on Maritime Search and Rescue, the 1974 International Convention for the Safety of Life at Sea (SOLAS), and the 1982 United Nations Convention on the Law of the Sea (UNCLOS) (3). Committed to this principle of “duty to rescue,” the NGOs aim to fill the gap left by national coast guards and international bodies. During the last few years, they have surely played a crucial role in SAR operations, saving countless lives. Nevertheless, the involvement of NGOs in SAR operations is an ongoing topic that sparks debates and controversies, particularly regarding their impact on migration patterns, the number of departures, and the number of deaths at sea.

## By the numbers

Around a dozen NGO vessels rescued over 110,000 migrants and refugees in distress in the Central Mediterranean Sea between 2014 and 2017, with an increase in their activity from about 1 thousand rescued in 2014 to more than 45.000 rescued per year in 2016 and 2017 (2, 4). Nevertheless, their operations have faced increasing scrutiny and criminalization by European Union member states (5).

Indeed, several European nations have alleged that these activities serve as a pull factor for migration, thereby indirectly endorsing illegal immigration. In response, some countries have halted NGO operations while others have shut the national ports to their ships, as also reported by the European Agency for Fundamental Rights (2, 6). Such measures, aligned with “non-entry” tactics (7), raise questions about their legality under the already cited international conventions. The “Land ahead,” which was once the primary reason for shouting at sea, has nowadays transformed into a disheartened “no end in sight,” a term used in a recent UNHCR report (8).

In the oscillation between viewing the sea as the old *Mare Nostrum* (our sea) and *Mare Nullius* (no one's sea) (6), recent statistical assessments with empirical data—finally—do not substantiate the claim that search-and-rescue operations are a contributing factor to irregular migration (9). This empirical evidence directly contradicts the NGOs intervention “pull factor” claim given that the difference between the prediction and observed series is far from statistically significant, while for the EU and Libya cooperation intervention period, the effect is slightly negative, which is suggestive of a reduction in the number of crossing attempts as a result of the coordinated pushbacks and the extension of the Libyan search-and-rescue zone, at what cost in terms of human rights we can just imagine.

## Heroes vs. criminals

The role of NGOs in migration and border governance is complex and multifaceted (10), and their relationship with the involved countries is particularly contentious. Over the years, Europe's Frontex (the European Border and Coast Guard Agency) has expanded and further solidified the externalization of European borders (11). However, the discontinuation of Italy's Mare Nostrum operation in 2014, which the European-led Joint Operation Triton replaced, represented a potentially more constrained approach to Search and Rescue (SAR) for migrants (5). This was followed by the even more limited Joint Operation Themis, which is currently ongoing. Alongside this, there is an ongoing effort to make agreements to block migrants before they embark on their sea journey, but with unstable governments or in states that still cannot Guarantee even minimal human rights.

In the past years, Libyan coastguards intercepted many individuals in the Central Mediterranean and returned them to Libya. Amid worsening political conditions in Libya, both the International Organization for Migration and the UN Refugee Agency renewed their appeal in July 2021, urging nations to avoid sending back anyone rescued at sea to Libya (12). Despite this, Italy extended its partnership agreement with Libya in July 2021. Malta established a memorandum of understanding with Libya in May 2020 to collaborate on countering irregular migration. At the same time, Malta waits for days to intervene when it is necessary (and legally required) (13).

Politics and media augmented the belief that NGOs may be aiding illegal migration or even directly collaborating with human traffickers and, as a result, NGOs have faced legal prosecution, and sometimes they lost social credibility (5, 6).

## Conclusions

The involvement of NGOs in SAR operations in the Mediterranean Sea has both positive and negative consequences. While NGOs have played a vital role in saving lives and providing humanitarian assistance, their operations have faced increasing scrutiny and criminalization. Governments all across Europe sometimes forget their legal obligation to do not betray some electoral expectations, using migration and NGOs for political reasons. However, SAR is a duty that human societies should not evade, and watching humans perish in those waters on television or in newspapers every day can lead to two outcomes: becoming desensitized or acting. NGOs, for better or worse, and which are not responsible at first for those images, seem to have chosen the latter. Drawing from the words of Cesare Pavese: “I'm unsure what realm in this sea's midst could be // But at the ocean's bottom, I'd rather not be.”
